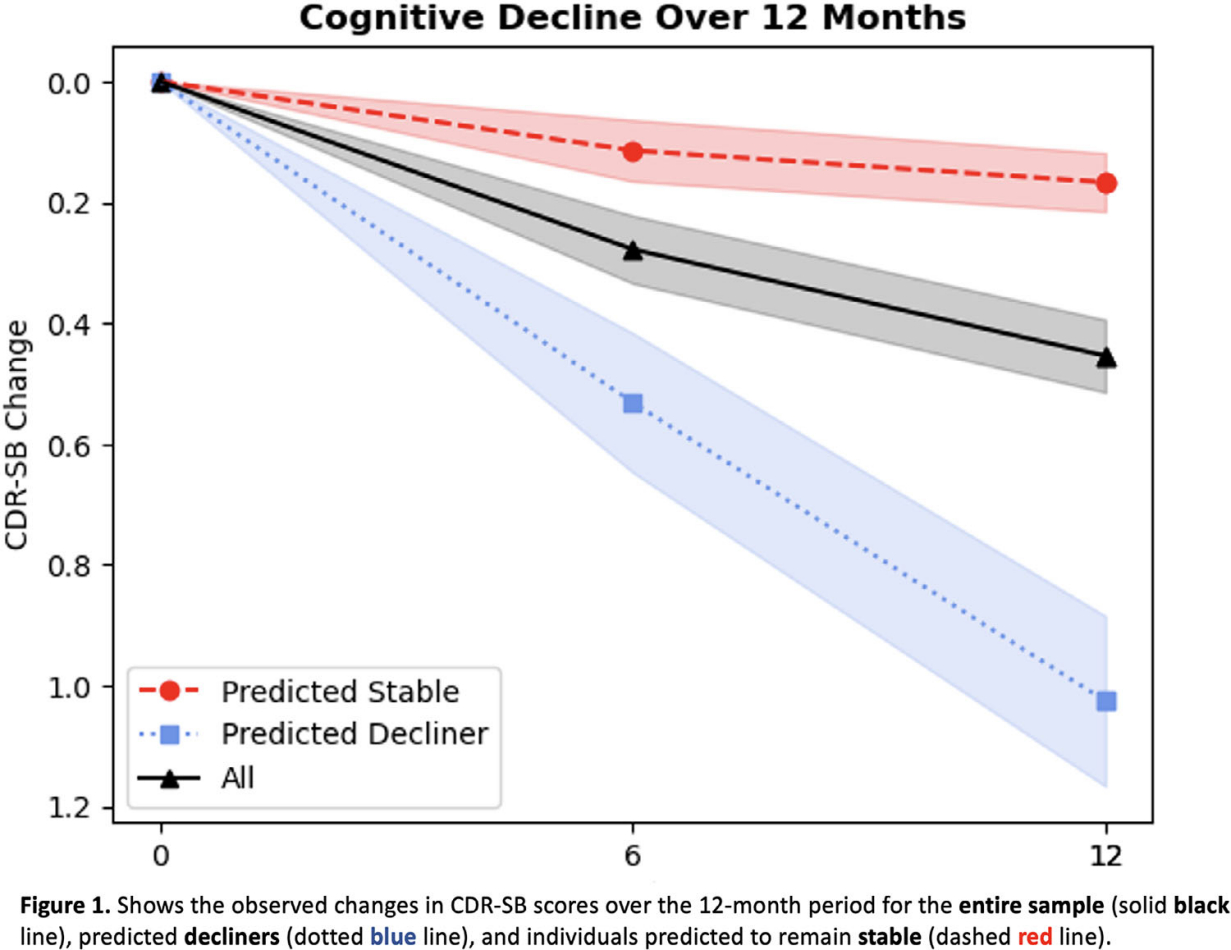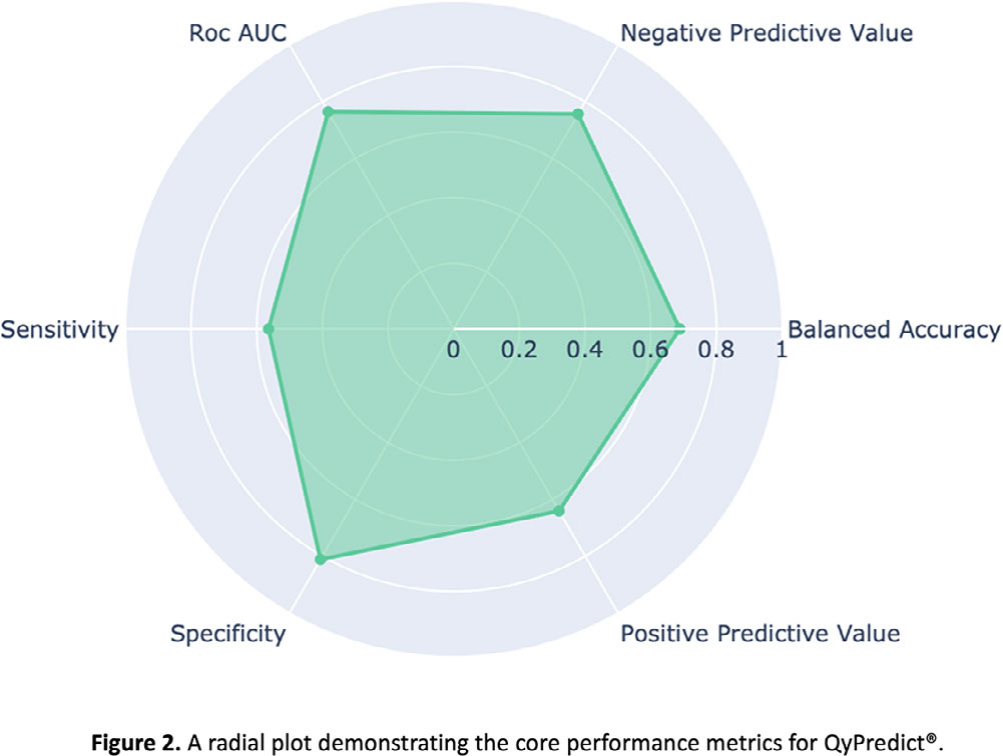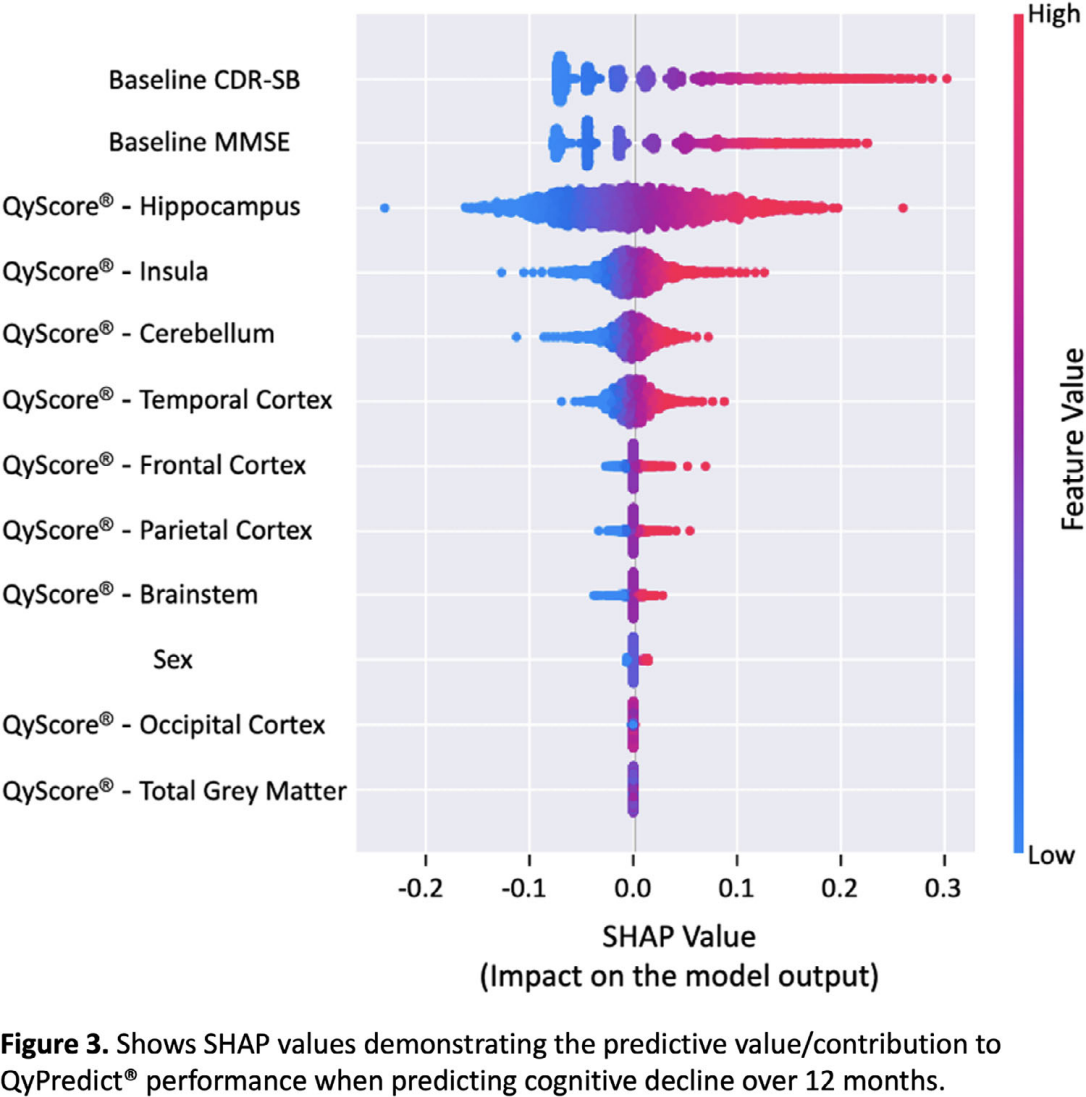# Qypredict®: A promising prognostic tool for anti‐amyloid therapy patient prioritization

**DOI:** 10.1002/alz.092232

**Published:** 2025-01-09

**Authors:** Luca Villa, Thomas Jubault, Nicolas Guizard, Elizabeth Gordon

**Affiliations:** ^1^ Qynapse, Paris France

## Abstract

**Background:**

The approval of anti‐amyloid therapies has led to a breakthrough in the treatment of Alzheimer’s disease. However, given these treatments remain expensive and are associated with significant side effects, identifying patients that would, and importantly would not, benefit from treatment is crucial in optimizing costs and minimizing health risks. QyPredict®, an advanced machine learning‐based predictive platform, has the potential to identify patients most likely to show cognitive decline, and may prove beneficial at prioritizing patient selection for therapies. Given limited healthcare resources, predictive tools need to perform well using only the most accessible and widely available clinical and demographic data and be trained on a broad and generalizable population.

**Method:**

Using data from ADNI, OASIS, and NACC, QyPredict® was applied to 1784 older individuals. To represent all individuals that may require future cognitive assessment, a variety of patient groups were included. The sample (mean and standard deviation (SD) age: 73.3(7.0), 42.2% female; baseline MMSE: 27.5(2.5)) was comprised of healthy controls (n=512), Alzheimer’s Disease patients (n=382), Mild Cognitive Impairment patients (n=660), and a mixture of other dementias, Parkinson’s disease, and psychiatric conditions (n=230). Cognitive decline was defined as an increase of at least 0.5 points on the CDR‐SB over 12 months. Model features included baseline MMSE, baseline CDR‐SB, sex, and regional grey matter volumes automatically derived using QyScore®, an FDA‐cleared and CE‐marked advanced neuroimaging platform.

**Result:**

Predicted decliners showed 5.8x greater cognitive decline on the CDR‐SB over 12 months compared to individuals predicted at baseline to remain stable (Predicted‐Decliner: 1.0(1.8) vs Predicted‐Stable: 0.17(0.85): Figure 1), which was statistically significant (t‐test: t(1782)=31.2, p<0.001). QyPredict® performed well, achieving the following: specificity: 0.81; sensitivity: 0.56; balanced accuracy: 0.69; negative predictive value: 0.76; ROC AUC: 0.77; positive predictive value: 0.64; F1 score: 0.6; precision: 0.62 (Figure 2). Baseline CDR‐SB, MMSE and QyScore®’s MRI markers of hippocampal and insular volumes demonstrated the greatest predictive value (Figure 3.)

**Conclusion:**

Using minimal clinical and demographic data, QyPredict® was able to accurately predict cognitive decline over a 12‐month period – regardless of diagnostic status. This may be invaluable when trying to prioritize which patients should receive anti‐amyloid therapies.